# Association of the prognostic nutritional index and overall survival in patients with colorectal cancer: A STROBE compliant retrospective cohort study

**DOI:** 10.1002/cam4.2212

**Published:** 2019-05-08

**Authors:** Julissa Luvián‐Morales, Sagrario González‐Trejo, José F. Carrillo, Roberto Herrera‐Goepfert, Vincenzo Aiello‐Crocifoglio, Dolores Gallardo‐Rincón, Francisco J. Ochoa‐Carrillo, Luis F. Oñate‐Ocaña

**Affiliations:** ^1^ Subdirección de Investigación Clínica Instituto Nacional de Cancerología (INCan) Mexico City Mexico; ^2^ Subdirección de Cirugía Instituto Nacional de Cancerología (INCan) Mexico City Mexico; ^3^ Departamento de Patología Instituto Nacional de Cancerología (INCan) Mexico City Mexico; ^4^ Departamento de Gastroenterología Instituto Nacional de Cancerología (INCan) Mexico City Mexico; ^5^ Departamento de Oncología Médica Instituto Nacional de Cancerología (INCan) Mexico City Mexico

**Keywords:** colorectal cancer, nutrition assessment, prognosis, prognostic nutritional index, survival

## Abstract

**Background:**

The TNM classification does not completely reflect the prognosis of patients with colorectal cancer (CRC). Several clinical factors have been used to increase its prognostic value, but factors pertaining to the patient's immunonutritional status have not usually been addressed. The aim of this study is to evaluate the role of Prognostic nutritional index (PNI) and other well‐known prognostic factors by multivariate analysis in a cohort of patients with CRC.

**Methods:**

This is a retrospective cohort study of consecutive patients with CRC managed in a cancer center between January 1992 and December 2016. Cox's model was used to define the association of the PNI and other factors with Overall survival (OS).

**Results:**

A total of 3301 patients were included: 47.7% were female and 52.3% were male, with a mean age of 58.7 years. By bivariate analysis, PNI was strongly associated with OS (Risk ratio [RR] 0.968, 95% Confidence interval [CI] 0.962‐0.974; *P* < 0.001). On multivariate analysis, PNI was an independent explanatory variable (as continuous variable and as categorized variable; RR 0.732, 95% CI 0.611‐0.878; RR 0.656, 95% CI 0.529‐0.813 and RR 0.646, 95% CI 0.521‐0.802, for quintiles 2, 3, and 4‐5, respectively); a biological gradient effect was demonstrated. The final prognostic model included PNI, location of the neoplasia in the colorectum, basal hemoglobin, lymphocyte count, neutrophil/lymphocyte ratio, platelet/lymphocyte ratio, TNM stage, differentiation degree, R classification, and postoperative complications.

**Conclusions:**

PNI is a significant and independent prognostic factor in patients with CRC. Its prognostic value adds precision to the TNM staging system including specific subgroups of patients with CRC; it should be evaluated in prospective clinical studies.

## 1 INTRODUCTION

Worldwide, colorectal cancer (CRC) is the third cause of cancer‐related mortality.^1^, ^2^ In Mexico, CRC places fourth and it is recognized as an increasing health problem.^1^ It is broadly acknowledged that surgery is the main curative option for patients with localized CRC and in the past few years, new treatments have improved overall survival (OS) in locally advanced or metastatic disease.^3^

The TNM staging classification is the most useful tool defining prognosis and guiding the tailoring of treatment options in CRC. However, OS cannot be completely explained by TNM stages or by the currently established prognostic factors.^4^ In CRC, several prognostic factors improving the prognostic value of the TNM classification have been described.^5^, ^6^, ^7^ However, more refined tools are needed to further increase their prognostic value. The Memorial Sloan‐Kettering Cancer Center model, which was designed to predict the 5‐ and 10‐year disease‐free survival after curative resection in patients with colon cancer, or the European CanCer Organisation/European Society of Surgical Oncology nomograms predicting recurrence, distant metastases and OS,^8^, ^9^ have been important contributions to the field. Nonetheless, the baseline immunonutritional status of patients was not considered as part of most of the conceptual models supporting these tools or were only defined in specific subpopulations of patients with CRC or specific stage groupings,^6^ even when inflammation has been widely recognized as the promoter of multiple hallmark functional components of cancer.^10^

Serum albumin is an important marker of nutritional status and the absolute blood lymphocyte count is a marker of immunologic function; both have been reported as good predictors of OS in CRC as well.^11^, ^12^ Together, as a Prognostic nutritional index (PNI), have demonstrated to be good predictors of complications after gastrointestinal surgery.^13^

The prevalence of malnutrition in patients with CRC ranges between 33% and 41% at the time of diagnosis,^14^ and is associated with an increased risk of developing complications and mortality.^15^ Also, malnutrition can increase hospital length of stay by up to 70%, thus increasing hospitalization costs by 23%^16^, ^17^; malnutrition is also associated with greater hematologic and nonhematologic toxicity due to chemotherapy and shorter progression‐free survival periods,^18^ hence, influencing the quality of life of patients, their functional capacities, and symptoms.^19^ Consequently, the aim of this study is to evaluate the prognostic role of PNI by multivariate analysis including other well‐known prognostic factors, and to develop a prognostic model in a cohort of patients with CRC treated in a tertiary care, cancer center.

## 2 MATERIAL AND METHODS

### 2.1 Patients

We included 3301 consecutive patients with CRC who were treated at the *“Instituto Nacional de Cancerología”* (INCan) in Mexico City, between January 1992 and December 2016. Inclusion criteria comprised a colonoscopy and biopsy establishing a final diagnosis of CRC and the histopathology had to be diagnostic of adenocarcinoma; the patient cohort included untreated female and male patients of any age. Chest X‐ray, liver ultrasonography, computed tomography, positron emission tomography scans, or magnetic resonance imaging were required for the staging protocol.

Data were extracted retrospectively from the clinical records and included the clinical history, physical examination, basal‐pretherapeutic blood cytology and blood chemistry, tumor markers, surgical procedures, adjuvant chemotherapy, radiation or chemoradiation, and diverse palliative procedures. The study protocol was designed according to the STROBE and AJCC criteria,^20^, ^21^ and it was approved by the Institutional Review Board and Bioethics Committee (Register number Rev/01/15).

### 2.2 Variables

The location of the neoplasm in the colorectum was established by colonoscopy, CT scan and/or laparotomy. Two independent pathologists reviewed the surgical pathology material, and disagreement was conciliated by consensus. The basal‐pretherapeutic serum albumin level and the basal‐pretherapeutic absolute lymphocyte count were recorded and the PNI was calculated as previously reported: ([serum albumin in g/dL × 10] + [0.005 × total lymphocyte count in cells/µL]).^13^

The seventh edition of the TNM staging system was used and patients treated before January 2010 were restaged.^22^ Surgery was coded as radical right or left hemicolectomy, radical sigmoidectomy, low anterior rectal resection, or abdominoperineal resection. Surgical morbidity was classified according to the Clavien‐Dindo classification.^23^ The number of lymph nodes retrieved, the number of positive lymph nodes and the metastatic lymph node ratio (number of positive/total lymph nodes retrieved) were calculated. Rectal cancer was treated following the total mesorectal excision approach; adjuvant chemotherapy, preoperative chemoradiation, or palliative chemotherapy were administered as appropriate, following the NCCN guidelines.^24^, ^25^

### 2.3 Statistical analysis

After descriptive analysis, the association of PNI with all clinically relevant variables was tested. Continuous variables were categorized by quintiles^26^; correlation analysis was obtained with Pearson's correlation coefficient; bivariate analysis of prognostic factors was performed with Student's *t* test, analysis of variance, or squared chi test, depending on whether the studied variables were continuous or categorical. The association of PNI with OS was analyzed using the Kaplan‐Meier method and differences were tested with the log‐rank test. Multivariate analysis was performed with the proportional hazards (Cox) model; Risk ratios (RR) were calculated as a measure of association along with their 95% Confidence intervals (CI). Interaction terms and proportionality assumptions were tested in the final model.^27^ Missing values were handled with the multiple imputation technique.^28^ Any probability of 0.05 or less was considered significant; 2‐tailed statistics were used in all cases, and computations were performed with the SPSS statistical software for Mac, version 23. (IBM Corp., Armonk, NY, USA).

## 3 RESULTS

### 3.1 Patients

There were 3301 patients with CRC that were included during the described study period; 1574 (47.7%) were female and 1727 (52.3%) were male, with a mean age of 58.7, (range 16‐97) (Standard deviation [SD] 15.6). The cohort included 8 patients (0.2%) living outside of the county; 1579 (47.8%) came from central Mexico (Mexico City, México, Morelos, Hidalgo, Tlaxcala, Puebla and Querétaro); 76 (2.3%) came from states in northern Mexico (Nayarit, Jalisco, Michoacán, San Luis Potosí, Guanajuato, Sinaloa, Sonora), and 122 (3.7%) proceed from southeast Mexico (Veracruz, Chiapas, Oaxaca, Tabasco and Yucatán).

Comorbidities were not recorded in 1466 cases (44.4%); systemic arterial hypertension was present in 342 (10.4%), diabetes mellitus in 240 (7.3%), and diverse chronic diseases in 1253 (38%). A Body mass index (BMI) of 25 or less were recorded in 2001 cases, 976 (29.6%) had a BMI from 25 to 30, and 324 (9.8%) had a BMI more than 30. The neoplasms were located as follows: 753 (22.8%) in the right colon, 118 (3.6%) in the transverse colon, 197 (6%) in the left colon, 551 (16.7%) in the sigmoid colon, and 1682 (51%) in the rectum.

Radical R0 resections were performed in 1818 patients (55.1%), R1 in 113 (3.4%) and 482 patients (14.6%) underwent R2 palliative resections; 888 patients (26.9) did not underwent surgical resection. Postoperative complications and mortality were recorded in 207 (8.6% of 2413 resected patients) and 34 cases (1.4% of 2413 resected patients), respectively.

The histopathology reports included the following diagnoses: 529 patients (16%) had well‐differentiated neoplasms, 2120 (64.2%) were moderately differentiated, 462 (14%) were poorly differentiated, and 190 (5.8%) were undifferentiated; 156 patients (4.7%) were classified as stage I, 947 (28.7%) as stage IIa, 87 (2.6%) as stage IIb, 149 (4.5%) were stage IIc, 98 (3%) were stage IIIa, 278 (8.4%) were stage IIIb, 553 (16.8%) were stage IIIc, 662 (20.1%) were stage IVa and 371 (11.2%) were stage IVb.

### 3.2 Associations with PNI

The absolute lymphocyte count and basal serum albumin presented poor correlation (*r* = 0.155; *P* < 0.0001) (Appendix Figure S1). Mean PNI was 44.45 (SD 8.16; range 11.6‐79), and its frequency distribution was normal (skewness −0.483, standard error [SE] 0.043; kurtosis 1.02, SE 0.085) (Appendix Figure S2).

The associations of the 5 categories of PNI with several clinical factors are listed in the Appendix Table S1. Age, TNM, location in the colorectum, R classification, use of neoadjuvant chemotherapy, use of neoadjuvant chemoradiation, BMI, adjuvant chemotherapy, and several laboratory parameters were associated with PNI.

### 3.3 Survival

Median OS of the cohort was 3.47 years (95% CI 3.01‐3.9). There were 1334 deaths (40.4%) during the follow‐up of this study. Median follow‐up time was 1.1 years (interquartile range: 0.29‐2.5 years).

Median OS for the 5 quintiles of PNI were 1.69 years (95% CI 1.34‐2.03), 2.68 (95% CI 1.92‐3.43), 3.69 (95% CI 2.67‐4.7), 5.11 (95% CI 3.87‐6.36), and 5.17 (95% CI 3.92‐6.42), respectively. The 5‐year OS rate for the 5 quintiles of PNI were 0.311 (SE 0.025), 0.409 (SE 0.028), 0.449 (SE 0.029), 0.518 (0.028) and 0.516 (0.028), respectively (*P* < 0.00001).

**Figure 1:**
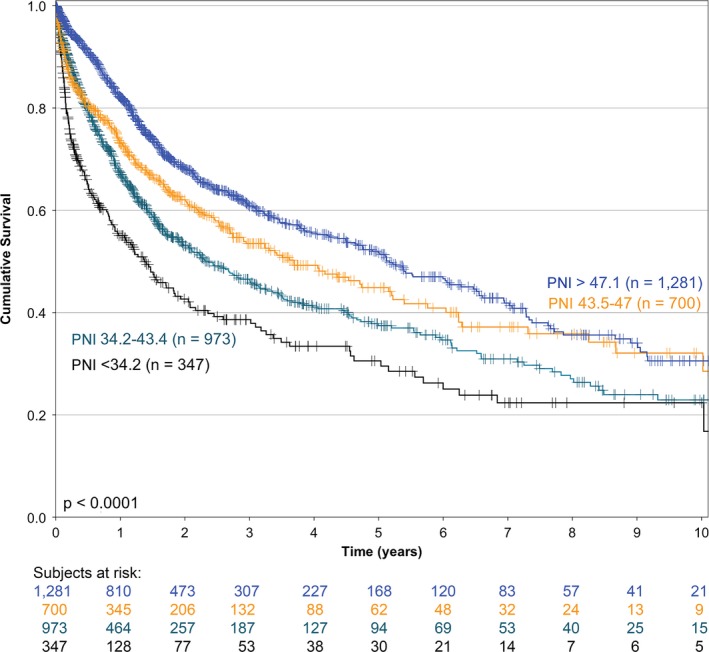
Kaplan‐Meier overall survival curves of all patients in the cohort according to the prognostic nutritional index (n = 3301)

**Figure 2:**
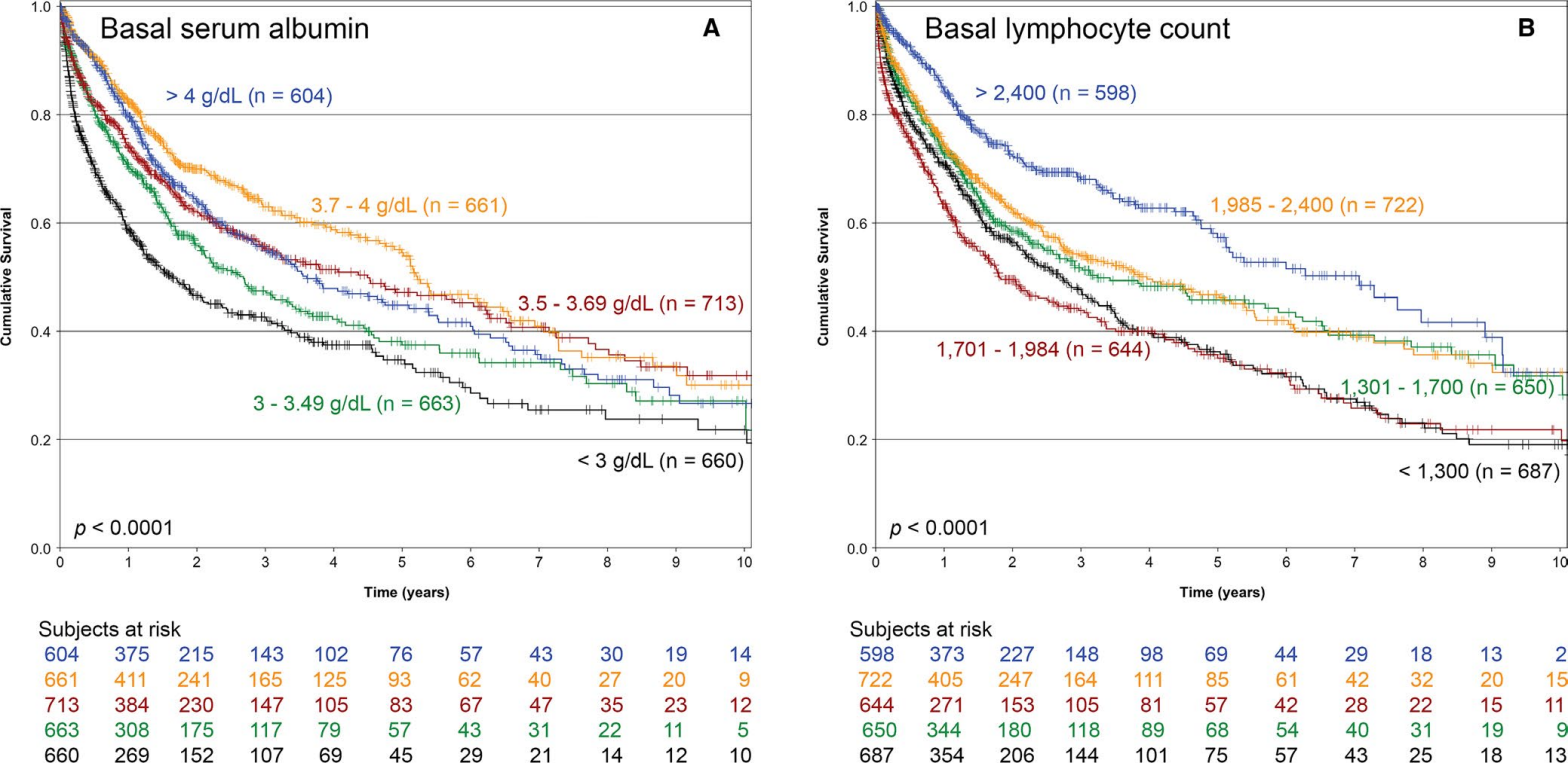
Kaplan‐Meier overall survival curves of all patients in the cohort according to (A) basal serum albumin level and (B) basal absolute lymphocyte count (n = 3301)

**Figure 3:**
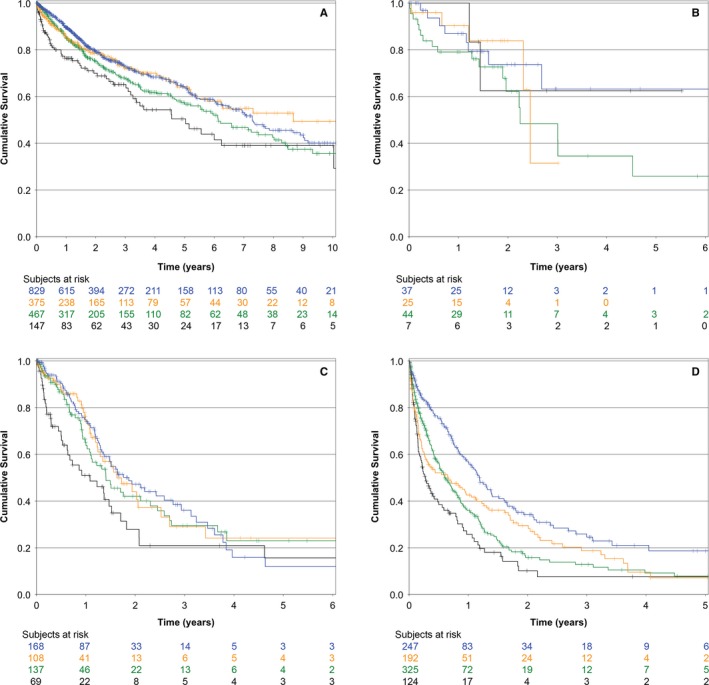
Kaplan‐Meier overall survival curves of all patients in the cohort according to the prognostic nutritional index across R classification strata (stratified analysis *P* < 0.0001). A, R0 surgical resection; B, R1 surgical resection; C, R2 surgical resection; D, no surgical resection performed. Blue line, first and second quintile; yellow line, third quintile; green line, fourth quintile; black line, fifth quintile

The bivariate association of several factors and OS is presented in Table 1. As presented in the table, when the PNI was divided in quintiles, it was strongly associated to OS. The fourth and fifth quintiles had the same prognosis, so these were analyzed as a single subgroup (Figure 1) (*P* < 0.0001), thereafter, only 4 groups were considered. The association of basal serum albumin and the absolute lymphocyte count with OS is depicted in Figure 2 (both associations *P* < 0.0001). The association of PNI and OS was conserved across R classification strata (stratified analysis *P* < 0.0001) (Figure 3).

**Table 1:** Bivariate association of selected prognostic factors and overall survival

Prognostic factor		RR	95% CI	*P*
Age		1	0.996‐1.003	0.804
Male gender		0.93	0.835‐1.035	0.184
Location	Right*	1	—	0.106
	Transverse	0.857	0.608‐1.207	0.377
	Left	1.04	0.811‐1.334	0.756
	Sigmoid	1.056	0.885‐1.259	0.548
	Rectum	1.163	1.013‐1.335	0.032
Basal hemoglobin		0.943	0.924‐0.962	<0.0001
Basal serum albumin		0.726	0.673‐0.784	<0.0001
Basal lymphocyte count		1	1‐1	<0.0001
PNI		0.968	0.962‐0.974	<0.0001
Carcinoembryonic antigen		1	1‐1	0.942
Neutrophil/lymphocyte count		1.038	1.029‐1.047	<0.0001
Platelet/lymphocyte count		1.709	1.493‐1.956	<0.0001
Neutrophil/platelet count		1.003	1.001‐1.005	0.003
Differentiation degree	Well*	1	—	<0.0001
	Moderate	1.344	1.14‐1.586	<0.0001
	Poor	1.882	1.53	<0.0001
	Undifferentiated	1.27	0.97‐1.664	0.082
TNM classification	I*	—	—	<0.0001
	IIa	3.064	1.823‐5.149	<0.0001
	IIb	3.386	1.733‐6.615	<0.0001
	IIc	8.604	4.958‐14.932	<0.0001
	IIIa	1.39	0.661‐2.922	0.385
	IIIb	2.575	1.477‐4.49	0.001
	IIIc	7.198	4.286‐12.088	<0.0001
	IVa	9.35	5.568‐15.701	<0.0001
	IVb	11.704	6.926‐19.779	<0.0001
R classification	R0*	1	—	<0.0001
	R1	1.454	1.027‐2.059	0.035
	R2	2.945	2.505‐3.462	<0.0001
	No surgical resection	5.792	5.104‐6.572	<0.0001
Surgical morbidity	grade 0*	1	—	0.655
	grade 1‐2	0.907	0.57‐1.445	0.682
	grade 3‐4	0.89	0.676‐1.171	0.405
Adjuvant chemotherapy		0.38	0.333‐0.434	<0.0001

CI, confidence interval; *p*, probability value; PNI, prognostic nutritional index; R, residual disease classification; surgical morbidity after Clavien‐Dindo classification; RR: risk ratio.

* Reference category.

The final multivariate model of prognostic factors associated with OS is shown with their estimators in Table 2.

**Table 2:** Final model of the multivariate analysis of prognostic factors associated with overall survival (*P* < 0.0001)

Prognostic factor		β (SE)	Exp β	95% CI	*P*
Location	Right*	—	1	—	0.08
	Transverse	−0.309 (0.177)	0.734	0.519‐1.037	0.08
	Left	0.077 (0.129)	1.08	0.838‐1.391	0.552
	Sigmoid	0.092 (0.093)	1.096	0.914‐1.315	0.321
	Rectum	−0.074 (0.075)	0.929	0.801‐1.077	0.33
Basal hemoglobin	(unit)	−0.027 (0.013)	0.973	0.949‐0.998	0.033
Lymphocyte	<1300 cells/mm^3^ *	—	1	—	<0.0001
	1300‐1700 cells/mm^3^	0.038 (0.1)	1.039	0.854‐1.265	0.701
	1701‐1980 cells/mm^3^	0.331 (0.108)	1.393	1.127‐1.721	0.002
	1981‐2400 cells/mm^3^	0.089 (0.114)	1.093	0.875‐1.367	0.433
	>2400 cells/mm^3^	−0.223 (0.138)	0.8	0.611‐1.049	0.106
Neutrophil/lymphocyte ratio	(unit)	0.031 (0.007)	1.031	1.018‐1.044	<0.0001
Neutrophil/lymphocyte ratio	<1.71*	—	1	—	0.047
	1.17‐2.34	−0.022 (0.103)	0.978	0.799‐1.196	0.828
	2.34‐2.79	0.236 (0.105)	1.266	1.031‐1.553	0.024
	2.8‐4	0.054 (0.111)	1.055	0.849‐1.312	0.628
	>4	0.122 (0.133)	1.13	0.871‐1.465	0.358
Platelet/lymphocyte ratio	<0.13*	—	1	—	0.1
	0.13‐0.17	−0.204 (0.096)	0.816	0.675‐0.985	0.034
	0.17‐0.22	−0.105 (0.102)	0.9	0.737‐1.101	0.305
	0.22‐0.31	−0.262 (0.111)	0.769	0.619‐0.957	0.019
	>0.31	−0.248 (0.134)	0.781	0.6‐1.015	0.064
PNI	<34*	—	1	—	<0.001
	34.1‐43.4	−0.311 (0.093)	0.732	0.611‐0.878	0.001
	43.5‐47	−0.422 (0.109)	0.656	0.529‐0.813	<0.0001
	>47	−0.436 (0.11)	0.646	0.521‐0.802	<0.0001
Differentiation degree	Well*	—	1	—	0.002
	Moderate	0.242 (0.085)	1.274	1.078‐1.506	0.005
	Poor/undifferentiated	0.352 (0.099)	1.421	1.169‐1.727	<0.0001
TNM classification	I*	—	1	—	<0.0001
	IIa	0.98 (0.266)	2.665	1.584‐4.484	<0.0001
	IIb	0.898 (0.344)	2.455	1.251‐4.817	0.009
	IIc	1.561 (0.285)	4.765	2.727‐8.325	<0.0001
	IIIa	0.2 (0.38)	1.222	0.58‐2.572	0.598
	IIIb	0.856 (0.285)	2.354	1.348‐4.112	0.003
	IIIc	1.551 (0.267)	4.715	2.793‐7.959	<0.0001
	IVa	1.228 (0.272)	3.416	2.004‐5.82	<0.0001
	IVb	1.369 (0.275)	3.932	2.292‐6.745	<0.0001
R classification	R0*	—	1	—	<0.0001
	R1	0.281 (0.18)	1.324	0.931‐1.883	0.118
	R2	0.895 (0.098)	2.446	2.018‐2.965	<0.0001
	No surgical resection	1.56 (0.082)	4.759	4.053‐5.589	<0.0001
Postoperative complication	(any)	0.25 (0.076)	1.284	1.106‐1.491	0.001

β, regression coefficient; CI, confidence interval of the beta exponent; exp β, beta exponent (corresponds to risk ratio); *p*, probability value; PNI, prognostic nutritional index; SE, standard error.

* Reference category.

## 4 DISCUSSION

The TNM classification is a construct of the three most robust prognostic factors and it is the basis when defining prognosis or tailoring different treatment options in CRC.^22^ Nevertheless, it has been recognized as inaccurate in defining prognosis in certain subsets of patients with CRC.^29^ Therefore, many prognostic factors have been reported with the goal of increasing the predictive value of the TNM classification. The AJCC has recognized that new prognostic factors should be included in the TNM classification with the intention of increasing its accuracy in estimating the prognosis of patients with cancer, and for this purpose, has stimulated the development of new prognostic models. Therefore, they are proposing the main criteria that are required for the design of useful and reliable prognostic models.^21^

The AJCC‐UICC have included other prognostic factors such as degree of differentiation, R classification, circumferential resection margin, tumor regression score, CEA level, lymphovascular invasion, perineural invasion, microsatellite instability, KRAS, NRAS, and BRAF.^22^

Most frequently evaluated prognostic factors include those directly associated to the neoplasm per se, that is, morphologic, proteomic, genomic or epigenomic, while the patient's immune and nutritional status have been often overlooked.^6^, ^7^, ^8^, ^9^ There is an increasing evidence that systemic inflammation plays a critical role in carcinogenesis and the invasion or metastatic processes in CRC.^30^ The tumor‐associated inflammatory response has been recognized to exert a contradictory effect, enhancing tumorigenesis and progression, and actually aiding incipient neoplasms to acquire hallmark capabilities.^10^ As part of those immunonutritional factors that can be measured in blood, PNI is thought to reflect patient's nutritional and immunological status and has been reported to be an important prognostic factor in several neoplasms such as gastric, hepatocellular, pancreatic, esophageal carcinoma, and malignant pleural mesothelioma.^31^ In the case of CRC, PNI has shown to be a robust prognostic factor for OS.^31^, ^32^

In this study, an important association between the PNI and OS in a large cohort of patients with CRC is reported. The final multivariate model strongly suggests that PNI is an independent explanatory variable, and it is relevant in conjunction with the neoplasm's location in the colorectum, TNM stage, differentiation degree, and several other well‐established prognostic factors. As recommended by AJCC, only OS was evaluated as significant outcome since we agree that the role of disease‐free survival or cancer‐specific mortality is controversial since the potential difficulty in reliably assigning the cause of death.^21^

The main pitfalls of this study are the retrospective nature of the data, the absence of C reactive protein values, the long‐time of recruitment, a validation protocol is lacking and the institutional reference bias inherent to any tertiary care medical institution. However, the main strengths of our findings are the large number of patients in the cohort and the prolonged follow‐up periods of most survivors. Serum albumin and absolute lymphocyte counts are inexpensive and readily available markers in any setting in almost all countries, so the PNI includes data obtained from a basic blood cytology and serum chemistry that are usually performed before any further diagnostic work‐up or therapeutic procedure is undertaken. The presented prognostic model can be easily constructed.

As far as we know, there are no reports that have used the PNI in a multivariate prognostic model in patients with CRC, and it has not been tested or validated in any nomogram or model. However, it has been used in other malignancies. In the case of CRC, an important association of PNI and OS after surgical resection has been reported as well as a comparison of systemic inflammatory and nutritional scores in CRC patients who underwent potentially curative resection.^15^ The same group has recently documented that PNI and the Glasgow prognostic score are independent explanatory variables by multivariate analysis, suppressing the prognostic value of the neutrophil/lymphocyte ratio (NLR) and neutrophil/platelet ratio (NPR).^33^ This is an important difference when compared with our findings because in our study, PNI does not annul the prognostic value of NLR, but only that of the NPR. Additionally, in the final model, the PLR was not concealed either (Table 2).

Figure 2 shows that the associations of basal serum albumin and basal absolute lymphocyte count with OS are very important. Both associations reveal an imperfect biological gradient, especially in the higher categories of both variables. Conversely, in Figure 1, the PNI presents an important biological gradient in its association with OS. The 2 higher quintiles of PNI were treated as one because both yielded a similar RR. These observations suggest that the construct of basal serum albumin and basal lymphocyte count is a much more accurate prognostic factor than the isolated values of either, considering the low bivariate correlation values (*r* = 0.155; Appendix Figure S1).

Several reports on CRC emphasize that a cut‐off value of PNI of 44.5 or more is associated to less frequent postoperative complications and a longer OS^15^, ^34^, ^35^, ^36^, ^37^; a PNI above 36 in patients who underwent elective low anterior resection with colorectal anastomosis, is associated with a longer disease‐free survival.^38^ Several cutoff points for the PNI are reported, usually range from 35 to 50.^32^

In our study, a cut‐off value for PNI is intentionally not defined, because the association of PNI and OS yielded a clear biological gradient by bivariate analysis (Figure 1). A forecast using the PNI as a continuous variable has enormous advantages from a statistical point of view. This allows the Cox's Proportional Hazards model to establish a much more accurate forecast model.

This is the first report on the association of PNI in CRC in Latin‐America and it is interesting to compare with the study of Sun et al^32^ which is a meta‐analysis performed mainly in Asian patients, including 6372 patients from ten centers. In our study, 3301 patients mainly from central Mexico were treated in a single cancer center.

The mechanism by which the PNI impacts prognosis is unknown. Serum albumin is produced mainly by hepatocytes and the albumin synthesis is regulated by several proinflammatory cytokines, which are produced by the host, tumor microenvironment and cancer cells, and may play crucial roles in carcinogenesis, cancer progression, and angiogenesis.^39^ The serum albumin level is correlated with an increased inflammatory response to the tumor.^39^ Lymphocyte cell subpopulations include CD4+ and CD8+ T‐cells, NK cells, NKT cells, gamma‐delta T‐cells, and B‐cells, which are closely related with tumor immunity. Therefore, the association between fewer lymphocytes and impaired tumor immunity has been reported.^40^ Serum albumin level and absolute lymphocyte count are regulated by related mechanisms.

In conclusion, immunonutritional parameters are relevant prognostic factors, and PNI must be measured and controlled in therapeutic interventional phase III studies in patients with CRC. PNI should be included in prognostic models to define OS in different subgroups of patients with CRC and its evaluation in predictive multivariate models including the TNM staging system is warranted. However, a validation study is required.

It is possible that improving the PNI with interventions designed to enhance the immunonutritional status, the surgical, chemotherapy or chemo‐radiation therapy morbidity may improve. Therefore, should be considered a significant component of the evaluation of new multimodal treatments in CRC.

Challenging problems in the near future would be to define the performance of PNI as a prognostic tool in specific subgroups of patients such as after complete resection of stage I/II colon cancer, the prediction of recurrence after adjuvant chemotherapy in stage III colon cancer, or in the prognosis of selected patients with resectable oligometastatic disease.

## CONFLICT OF INTEREST

All authors state that there are no financial or nonfinancial competing interests.

## AUTHORS' CONTRIBUTIONS

JLM, SGT, JFC: Designed the study, construct the database, conducted the study and follow‐up of patients, and analyzed and interpreted the patient data. JLM, SGT, and RHG: Designed the study, performed the histological examination of slides, wrote and edited the final version of the manuscript. VAC, DGR, FJOC, and LFOO: Designed the study, wrote the study protocol, conducted the study, analyze and discuss results, wrote and edited the final version of the manuscript. JLM, LFOO: data curation and statistical analysis, and were the major contributors in writing the manuscript. All authors read and approved the final manuscript.
