# Supplementary figures and images for: Association of the prognostic nutritional index and overall survival in patients with colorectal cancer: A STROBE compliant retrospective cohort study
Source: Cancer Med. 2019 May 8;8(7):3379–88. doi: 10.1002/cam4.2212 (PMC6601598; doi:10.1002/cam4.2212)

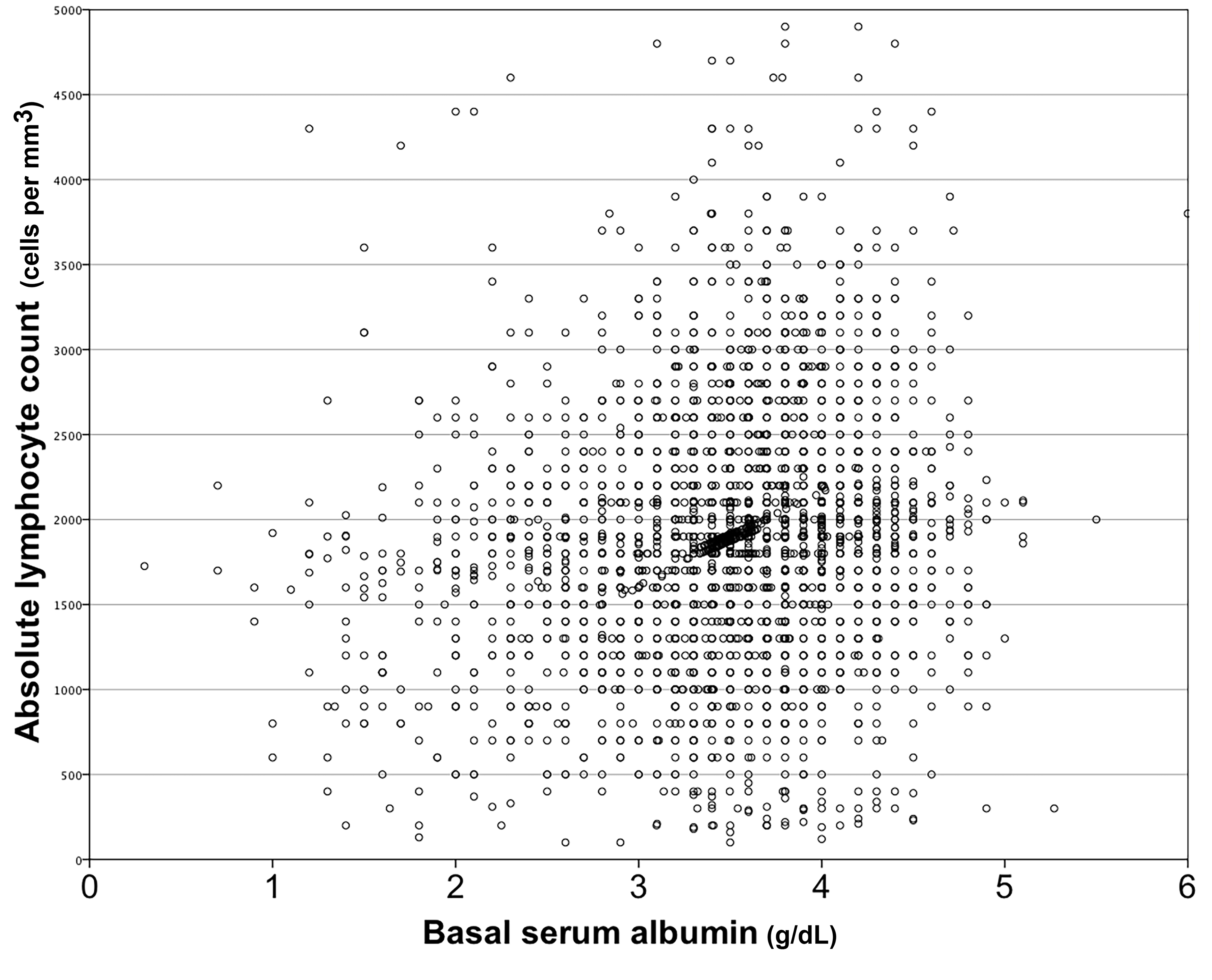

Supplement: Supplementary file 1 [file CAM4-8-3379-s001.tif]

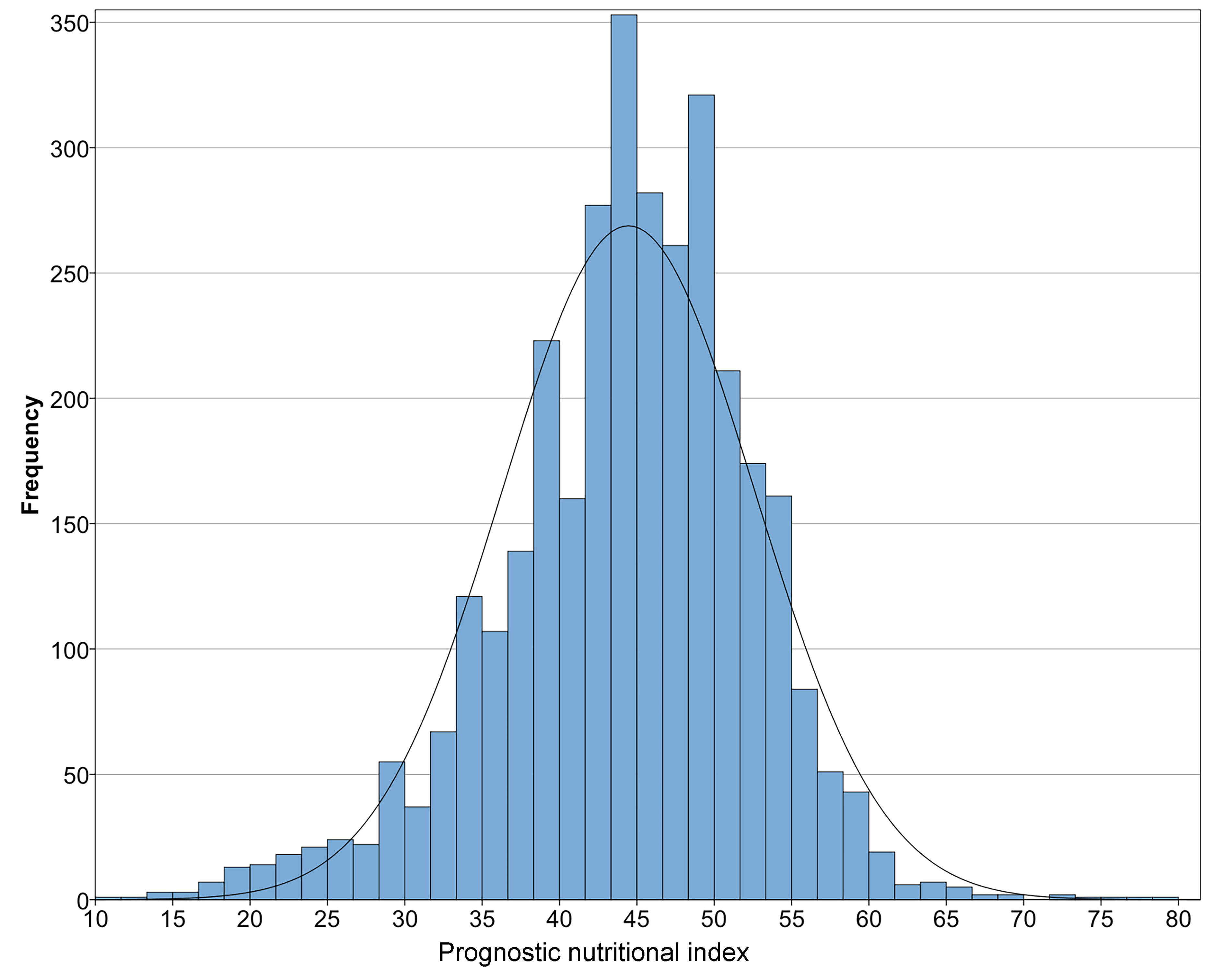

Supplement: Supplementary file 2 [file CAM4-8-3379-s002.tif]
