# Supplementary material for: Association of the prognostic nutritional index and overall survival in patients with colorectal cancer: A STROBE compliant retrospective cohort study
Source: Cancer Med. 2019 May 8;8(7):3379–88. doi: 10.1002/cam4.2212 (PMC6601598; doi:10.1002/cam4.2212)
Supplement: Supplementary file 3 [file CAM4-8-3379-s003.docx]

**Complementary Table A.** Prognostic Nutritional Index values of patients and their association with selected clinical variables described in the study.

| Factor | <38.5 (*n*=705) | 38.6-43.4 (*n*=615) | 43.5-47  (*n*=700) | 47.1-51 (*n*=644) | >51  (*n*=637) | *p* |
| --- | --- | --- | --- | --- | --- | --- |
| Age ^*^ | 60.2 (16.2) | 61.5 (16.5) | 58.6 (14.7) | 58 (15) | 55.13 (1) | **<0.0001** |
| Gender ^†^  Women  Men | 367 (52)  338 (48) | 293 (47.6)  322 (52.4) | 324 (46.3)  376 (53.7) | 304 (47.2)  340 (52.8) | 286 (44.9)  351 (55.1) | 0.092 |
| TNM classification ^†^  I  IIa  IIb  IIc  IIIa  IIIb  IIIc  IVa  IVb | 19 (2.7)  175 (24.8)  15 (2.1)  46 (6.5)  10 (1.4)  41 (5.8)  106 (15.1)  183 (28.1)  108 (26) | 23 (3.7)  151 (24.7)  17 (2.8)  38 (6.2)  17 (2.8)  33 (5.4)  107 (17.5)  144 (23.6)  80 (13.1) | 34 (5)  189 (28)  16 (2.4)  28 (4.1)  30 (4.5)  73 (10.8)  121 (17.9)  127 (18.8)  56 (8.3) | 33 (5.7)  213 (36.7)  14 (2.4)  19 (3.3)  23 (3.9)  62 (10.7)  103 (17.7)  114 (19.6)  56 (9.6) | 46 (7.2)  219 (34.6)  24 (3.8)  18 (2.8)  18 (2.8)  67 (10.6)  109 (17.2)  84 (13.3)  47 (7.4) | **<0.0001** |
| Baseline CEA ^*^ | 126 (997) | 136 (777) | 331 (4063) | 302 (2391) | 148 (859) | 0.267 |
| Location ^†^  right  transverse  left  sigmoid  rectum | 219 (31.1)  38 (5.4)  36 (5.1)  96 (13.6)  316 (44.8) | 132 (21.5)  24 (3.9)  42 (6.8)  95 (15.4)  322 (52.3) | 148 (19.7)  20 (2.8)  43 (6.1)  121 (17.2)  368 (52.6) | 118 (18.3)  23 (3.6)  41 (6.3)  122 (18.9)  340 (52.8) | 136 (21.4)  13 (2)  35 (5.5)  117 (18.4)  336 (52.7) | **<0.0001** |
| Differentiation degree ^†^ well  moderate  poor  undifferentiated | 101 (14.3)  454 (64.4)  114 (16.2)  36 (5.1) | 98 (15.9)  397 (64.5)  86 (13.9)  34 (5.5) | 106 (15.1)  468 (66.9)  95 (13.6)  31 (4.4) | 106 (16.6)  421 (65.4)  81 (12.6)  36 (5.6) | 118 (18.5)  380 (59.7)  86 (13.5)  53 (8.3) | **0.055** |
| “R” Classification ^†^  R0  R1  R2  No surgical resection | 308 (44.2)  23 (3.3)  125 (17.9)  241 (34.6) | 306 (51.2)  28 (4.7)  81 (13.5)  183 (30.6) | 375 (54.9)  25 (3.7)  108 (15.8)  175 (25.6) | 400 (64.3)  22 (3.5)  83 (13.3)  117 (18.8) | 429 (68.8)  15 (2.4)  85 (13.6)  95 (15.2) | **<0.0001** |
| Neoadjuvant radiotherapy ^†^ | 95 (13.9) | 104 (17.6) | 116 (17.5) | 136 (22) | 125 (20.4) | **0.002** |
| Neoadjuvant chemotherapy ^†^ | 97 (14.2) | 105 (17.8) | 119 (18) | 142 (22.9) | 132 (21.6) | **<0.0001** |
| Hemoglobin ^*^ | 10.6 (2.5 | 11.7 (2.2) | 12.5 (2) | 13.2 (2.2) | 13.7 (2.2) | **<0.0001** |
| NLR ^*^ | 5.5 (6.2) | 4 (4.3) | 2.9 (2) | 2.3 (1.2) | 1.95 (0.9) | **<0.0001** |
| PLR ^*^ | 0.42 (0.44) | 0.28(0.22) | 0.21(0.11) | 0.17 (0.07) | 0.13 (0.06) | **<0.0001** |
| Platelet count ^*^ | 437 (178) | 386 (140) | 361.7(121) | 340 (123) | 328 (107) | **<0.0001** |
| NPR ^*^ | 15.9 (28) | 14.6 (7.9) | 14.9 (12.2) | 15.7 (25.4) | 15.3 (8.2) | 0.723 |
| Neutrophil count ^*^ | 5.5 (2.3) | 5.1(1.7) | 4.8 (1.6) | 4.6 (1.7) | 4.5 (1.4) | **<0.0001** |
| Monocyte count ^*^ | 0.66 (0.36) | 0.67 (0.31) | 0.67 (0.29) | 0.59 (0.25) | 0.64 (0.46) | **<0.0001** |
| Globulin ^*^ | 3.9 (0.9) | 3.8 (0.8) | 3.9(0.8) | 3.5 (0.7) | 3.4 (0.6) | **<0.0001** |
| Albumin/globulin ratio ^*^ | 0.69 (0.24) | 0.92 (0.49) | 0.97 (0.27) | 1.14 (0.25) | 1.26 (0.3) | **<0.0001** |
| Body Mass Index ^*^ | 23.2 (4.3) | 24 (3.8) | 24.5 (4.1) | 25 (4.2) | 25.4 (4.2) | **<0.0001** |
| Adjuvant chemotherapy^†^ (yes) | 159 (22.8) | 153 (25.5) | 202 (30.1) | 214 (34.2) | 213 (34.2) | **<0.0001** |
| Radiotherapy^†^  (yes) | 29 (4) | 36 (6) | 39 (5.9) | 45 (7.2) | 47 (7.6) | 0.084 |
| Palliative chemotherapy^†^ (yes) | 118 (16.9) | 113(18.9) | 110 (16.5) | 99 (15.8) | 85 (13.7) | 0.169 |
| Palliative radiotherapy^†^  (yes) | 30 (4.3) | 16 (2.7) | 20 (3) | 21 (3.3) | 13 (2.1) | 0.207 |

CEA, carcinoembryonic antigen; R, residual disease classification, NLR, neutrophil/lymphocyte ratio, PLR, platelet/lymphocyte ratio; NPR, neutrophil/platelet ratio; values represent absolute (percentage); ^*^, represent continuous variables and numbers are, mean (standard deviation).
